# Effects of prolonged use of virtual reality smartphone-based head-mounted display on visual parameters: a randomised controlled trial

**DOI:** 10.1038/s41598-021-94680-w

**Published:** 2021-07-28

**Authors:** Hyeon Jeong Yoon, Hyun Sik Moon, Mi Sun Sung, Sang Woo Park, Hwan Heo

**Affiliations:** Department of Ophthalmology, Chonnam National University Medical School and Hospital, 42 Jebong-ro, Dong-gu, Gwangju, 61469 South Korea

**Keywords:** Eye diseases, Public health

## Abstract

We investigated the effects of using a virtual reality smartphone-based head-mounted display (VR SHMD) device for 2 h on visual parameters. Fifty-eight healthy volunteers were recruited. The participants played games using VR SHMD or smartphones for 2 h on different days. Visual parameters including refraction, accommodation, convergence, stereopsis, and ocular alignment and measured choroidal thickness before and after the use of VR SHMD or smartphones were investigated. Subjective symptoms were assessed using questionnaires. We analyzed the differences in visual parameters before and after the use of VR SHMD or smartphones and correlations between baseline visual parameters and those after the use of the devices. Significant changes were observed in near-point convergence and accommodation, exophoric deviation, stereopsis, and accommodative lag after the use of VR SHMD but not after that of smartphones. The subjective discomfort associated with dry eye and neurologic symptoms were more severe in the VR group than in the smartphone group. There were no significant changes in refraction and choroidal thickness after the use of either of the two devices. The poorer the participants’ accommodation and convergence ability the greater the resistance to changes in these visual parameters, and participants with a large exophoria were more prone to worsening of exophoria than those with a small exophoria.

## Introduction

Among the different virtual reality (VR) devices, head-mounted display (HMD) is an immersive medium that creates for the users the sensation of being transported into a virtual three-dimensional (3D) world. The VR market is expected to reach 18.9 billion US dollars in entertainment by 2025, with the gaming market accounting for one-third of all VR content^1^. The proportion of individuals using VR devices for gaming and education has increased. However, only a few studies have investigated the effects of VR HMD on the eyes^1–5^.

Ha et al.^3^ investigated the clinical effects of watching VR HMD movies for 30 min, and with the exceptions of transient refractive error and binocular alignment, no significant clinical changes were found. Turnbull et al.^4^ reported that the choroid was thickened and that binocular status indices, such as gaze stability, stereopsis, and accommodative amplitudes, did not change after the use of indoor- and outdoor-environment VR HMD for 40 min. VR HMD is mainly used to play games in immersive conditions, which constantly demands vergence adaptation, and are associated with a conflict between accommodation and convergence. However, these studies did not use immersive content, such as games. Our previous study showed that there was no change in refraction after the use of VR HMD, but the decrease in the ability of accommodation and convergence was higher after the use of a VR HMD in an immersive mode than after the use of a non-immersive mode for 30 min^5^. According to health and safety warnings provided by the manufacturers, continuous use of VR HMD for > 30 min is not recommended^6^. However, the average duration of VR HMD sessions for entertainment in the US in 2018 was 38 min; in fact, users of Sony PlayStation VR spent a mean of 49 min of playing time per VR session^2^. Although a few studies have reported that viewing stereoscopic images on 3D devices may induce visual asthenopia such as visual discomfort and fatigue^3,4,7–9^, the effects of using newer types of VR HMD for hours have not been investigated.

We hypothesized that the use of VR HMD for a long time could affect visual parameters such as refraction. We conducted this study to investigate the effects of using VR smartphone-based HMD (SHMD) for 2 h on visual parameters, including refraction, accommodation, convergence, stereopsis, and ocular alignment, as well as on choroidal thickness and subjective symptoms.

## Methods

Fifty-eight healthy volunteers aged 20–39 years, who had 20/20 or better best-corrected visual acuity, were recruited (01/12/2017–28/02/2018). The participants had no ophthalmologic disorder, including amblyopia, presbyopia, or corneal or retinal disease and no history of ocular surgery. The participants used a VR SHMD and a smartphone each for 2 h; the purpose of the latter was to serve as a control. Half of the randomly selected subjects used VR SHMD first, and the remaining participants used smartphones first. One experiment (VR and smartphone use) was performed over 2 days with 1-day intervals. Informed consent was obtained from all 58 volunteers who were enrolled in the study. Ethics committee approval was obtained from the Chonnam National University Hospital Institutional Review Board (Gwangju, Korea). The study protocol adhered to the guidelines of the Declaration of Helsinki. We registered this clinical study to an International Standard Randomized Controlled Trial Number (ISRCTN 48251379) (20/02/2021).

### Devices

Samsung Gear VR SHMD (Gear VR Innovator Edition; Samsung, Suwon, Korea) were used in the study. Headsets were assembled with a smartphone (Galaxy S6; Samsung) with 1440 × 2560 resolution and 60 Hz refresh rate. A fixed-degree convex lens was positioned in front of each eye. The distance between the screen and the front of the subject’s eyes was approximately 50 mm to 65 mm. A control wheel at the top of the headset enabled the participants to adjust the inter-pupillary distance.

The participants used the devices while seated on a freely rotating chair. For the VR experiment, the participants freely played a VR game (Lands End, Ustwo Games, UK) that was graded as “comfortable” on a platform provided by Oculus for 2 h^10^. For the smartphone experiment, the participants used a smartphone with a 5.1-inch light-emitting diode screen from the same manufacturer (Samsung) to play a game (Tetris, Electronic Arts, CA, USA) for 2 h. If the participants experienced discomfort while using the device, they were allowed a 5-min rest that was not included in the 2-h of playtime.

### Measurement of refraction and accommodation

Refraction and accommodation were measured using a binocular open-field refractor (Auto Ref/Keratometer WAM-5500, Grand Seiko Co. Ltd, Hiroshima, Japan). Refraction was measured with or without glasses. The spherical equivalent (sphere + 1/2 of the cylinder) was used for the calculation. The accommodative amplitude was calculated by subtracting the refractions obtained, under monocular condition, while viewing a 1 cm × 1 cm E-shaped target at a distance of 20 cm from those obtained while viewing the target at a distance of 5 m. The accommodative lag was calculated using the value by which the participant’s refraction differed from the accommodative stimulus. In this study, the value was calculated by subtracting − 5.0 diopter (D) from the refractions obtained with the target at a distance of 20 cm.

### Measurements of other visual parameters

Monocular near-point accommodation (NPA) was obtained using Donder’s push-up method. A 20/30 single letter on a fixation stick positioned approximately 50 cm from the subject served as the target, and it was moved gradually closer to the subject at a rate of approximately 5.0 cm/s until the subject noticed the blurring of the target.

The near-point of convergence (NPC) was also obtained. The fixation target, the starting point of the examination, and moving velocity of the fixation target were the same as those previously described for the NPA measurement. The first point at which the corneal reflex of the participants began to extend outward was considered the endpoint.

Stereopsis was measured using a near stereopsis vision test (Stereo Fly SO-001 test; Stereo Optical Co., Chicago, IL, USA). Stereopsis of 2500–1200 s of arc, 800–40 s of arc, and 400–100 s of arc was measured using fly photos, graded circle test, and animal test for children, respectively. The test stereogram was held at a distance of 40 cm from the subject during the test. The threshold stereopsis level was recorded in seconds of arc. To facilitate statistical analyses and calculation of means and differences, we used logarithmic transformation of stereopsis, with natural logs.^11^.

The presence and magnitude of ocular deviations at far (5 m) and near (33 cm) distances were verified using the cover test and alternating cover test with a prism. A standard set of loose plastic prisms was used for all measurements. The individual prisms increased in power from 1 to 10 prism diopter (PD) in 1-PD increments and 0–20 PD in 2-PD increments.

Ocular dominance was determined using the hole-in-the-card test, in which the subject was asked to hold a card with a hole at arm’s length and to focus, with both eyes, on an object placed 3 m away. The examiner then alternately occluded the eyes to determine the dominant eye, i.e., the eye that was viewing the object through the hole.

All visual parameters were measured, in the order listed above, before and after the participants played games using the VR SHMD or smartphone games. The visual parameters were measured after a 3-min break with the eyes closed after the use of the VR or smartphone. All measurements were repeated three times for each tested eye, and the results were reported as mean values. All parameters were examined by a single examiner (HJY).

### Choroidal thickness

We used a Heidelberg Spectralis with 870-nm wavelength (Heidelberg Engineering, Heidelberg, Germany) to obtain spectral-domain optical coherence tomography images of the posterior segment of the eye. The choroid was imaged using the enhanced depth imaging modality with eye-tracking and automated real-time averaging features. The scan through the fovea should have a prominent specular reflex at the bottom of the foveal pit.

Choroidal thickness was measured using the Heidelberg Eye Explorer software (Heidelberg Engineering, Heidelberg, Germany) (Version 1.9.10.0) provided by the instrument manufacturer. We used the semiautomatic segmentation method for choroidal thickness measurement^12^. We manually selected a new line at the choroid–scleral border (CSB). We retained the automatically defined Bruch’s membrane (BM) line, and the software calculated the vertical distance between the two segmentation lines. The choroidal thickness was defined as the vertical distance between the BM and CSB. The five targets were divided into 1- and 3-mm zones from the fovea. The choroidal thickness measurements were performed by a single trained examiner masked to the participants’ data (HJY).

### Evaluation of subjective symptoms

The following 13 symptoms were included in the questionnaire that was based on a computer vision syndrome questionnaire, which was previously reported by Seguí Mdel et al.^5,12^: dry eye symptoms (burning, feeling of a foreign body, excessive blinking, tearing, dryness, tingling, and increased sensitivity to light), visual disturbance (blurred vision, double vision, and difficulty focusing for near vision), and neurological symptoms (headache, dizziness, and nausea). The symptom sensation questionnaire included six identical analog scales (0 = none to 6 = too severe to tolerate), and the subject recorded the magnitude of each symptoms compared relative to that at the baseline. After playing the VR and smartphone games, the participants completed the questionnaire.

### Statistical analysis

Statistical analyses were performed using SPSS version 18.0 (IBM Corporation, Armonk, NY, USA) for Windows (Microsoft Corporation, Redmond, WA, USA). The distributions of all variables were assessed using the Shapiro–Wilk test. Normally distributed data are presented as mean ± standard deviation, whereas data with non-normal distribution are presented as median [interquartile range]. Variables showing normal distribution were analyzed using the paired t-test, and those with non-normal distribution were analyzed using the Wilcoxon signed-rank test. Differences in subjective symptoms between the devices were compared using the Wilcoxon signed-rank test. Spearman’s correlation test was used to evaluate the bivariate correlation between baseline data (refractive error, accommodative amplitude and lag, NPA, NPC, and ocular deviation) and the changes in these parameters after the use of VR. The variables of a single eye, including refraction, NPA, and accommodative parameters, were solely correlated with those of the corresponding eye. For all tests, *p* < 0.05 was considered statistically significant. If the data were not normally distributed, the Benjamini–Hochberg procedure using a false discovery rate of 0.25 was applied. The sample size was calculated using a priori power analysis for the Wilcoxon signed-rank test (matched pairs) in G*Power software (version 3.1.9.4; Heinrich-Heine University, Germany) with a level of α = 0.05, power of 95%, and an effect size of 0.5. The power and effect size were determined to detect a 0.5 diopter difference in refractive power, based on our previous study. Accordingly, a sample size of 47 patients was found to be sufficient. Therefore, we recruited a total of 58 participants considering the drop-out rate of 20%.

## Results

Fifty-eight participants aged 25.2 ± 3.9 years were included. The mean refractive errors of the right and left eyes were − 3.56 ± 2.40 D and − 3.24 ± 2.34 D, respectively. Five participants took a 5-min rest from playing the VR games because of mild headache or nausea; however, they completed the experiment.

**Figure 1:**
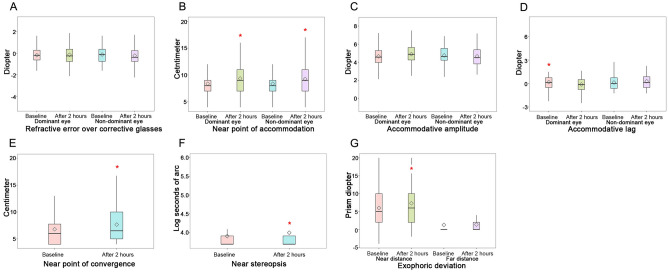
Changes in visual parameters after a 2-h virtual reality gaming session; (**A**) refraction with glasses, (**B**) near point of accommodation (**C**) accommodative amplitude, (**D**) accommodative lag, (**E**) near-point convergence, (**F**) near stereopsis, and (**G**) exophoric deviation at the near and far distances.

After playing the VR games, the mean refractive error over corrective glasses for both eyes did not change significantly (Pre: − 0.125 [1.13] D, Post: − 0.250 [0.94] D in the dominant eye [*p* = 0.540]; Pre: − 0.125 [1.13] D, Post: − 0.375 [1.00] D in the non-dominant eye [*p* =  0.528]). The NPA receded in the dominant eye (Pre: 8.00 [2.00] cm, Post: 9.00 [4.00] cm; *p* < 0.001) and the non-dominant eye (Pre: 8.00 [2.00] cm, Post: 9.00 [4.00] cm; *p* = 0.001). There were no changes in the accommodative amplitude (Pre: 4.65 ± 1.05 D, Post: 4.89 ± 1.10 D in the dominant eye [*p* = 0.062]; Pre: 4.75 ± 1.00 D, Post: 4.54 ± 1.53 D in the non-dominant eye [*p* = 0.708]). The accommodative lag in the dominant eye changed to accommodative lead after the use of VR SHMD (Pre: 0.19 ± 1.05 D, Post: − 0.11 ± 0.93 D; *p* = 0.030), and the accommodative lag remained unchanged in the non-dominant eye (Pre: 0.13 ± 0.93 D, Post: 0.19 ± 1.05 D; *p* = 0.924). The NPC receded (Pre: 6.00 [4.00] cm, Post: 6.50 [5.25] cm; *p* < 0.001), and near stereopsis worsened (Pre: 3.69 [0.22] logArcsec, Post: 3.69 [0.22] logArcsec; *p* = 0.005). There were no significant changes in the angle of exophoric deviation at a far distance (Pre: 0.00 [0.00] PD, Post: 0.00 [2.00] PD; *p* = 0.143); however, the angle of exophoric deviation at a near distance increased (Pre: 5.00 [9.00] PD, Post: 6.00 [9.00] PD; *p* < 0.001; Fig. 1).

**Figure 2:**
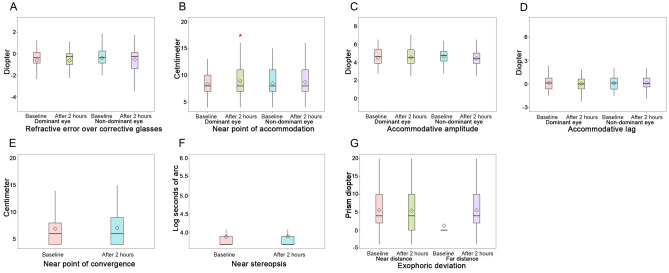
Changes in visual parameters after a 2-h smartphone gaming session; (**A**) refraction with glasses, (**B**) near point of accommodation (**C**) accommodative amplitude, (**D**) accommodative lag, (**E**) near-point convergence, (**F**) near stereopsis, and (**G**) exophoric deviation at the near and far distances.

After the smartphone gaming sessions, the mean refractive error over glasses for both eyes did not change (Pre: − 0.375 [1.13] D, Post: − 0.250 [1.00] D in the dominant eye [*p* = 0.883]; Pre: − 0.375 [1.25] D, Post: − 0.250 [1.56] D in the non-dominant eye [*p* = 0.152]). Only the NPA in the dominant eye receded after smartphone gaming use (Pre: 8.00 [3.50] cm, Post: 8.00 [4.00] cm; *p* = 0.002). However, there were no changes in the NPA in the non-dominant eye (Pre: 8.00 [4.00] cm, Post: 8.00 [4.00] cm; *p* = 0.071). In addition, there were no significant changes in the accommodative amplitude (Pre: 4.49 ± 1.02 D, Post: 4.53 ± 1.23 D in the dominant eye [*p* = 0.795]; Pre: 4.66 ± 0.95 D, Post: 4.44 ± 1.06 D in the non-dominant eye [*p* = 0.106]) and accommodative lag (Pre: 0.125 [1.38] D, Post: 0.000 [1.25] D in the dominant eye [*p* = 0.670]; Pre: 0.125 [1.47] D, Post: 0.063 [1.13] D in the non-dominant eye [*p* = 0.716]). The NPC (Pre: 6.00 [4.00] cm, Post: 6.50 [5.25] cm), near stereopsis (Pre: 3.69 [0.22] logArcsec, Post: 3.69 [0.22] logArcsec), and exophoric deviation at the near and far distances (Pre: 0.00 [0.00] D, Post: 0.00 [2.00] PD at the far distance; Pre: 4.00 [9.00] PD, Post: 4.00 [10.00] PD at the near distance) were also unchanged (*p* = 0.194, *p* = 0.172, *p* = 1.000, and *P* = 0.616, respectively; Fig. 2).

Table 1 shows the subjective symptoms that were reported regarding the use of the two devices. Visual disturbances such as difficulty focusing for near vision and neurological symptoms such as headache, dizziness, and nausea were more severe during use of the VR SHMD use than during smartphone use (all ps ≤ 0.013). In addition, dry eye symptoms including burning, feeling of a foreign body, and tearing were more severe after use of the VR SHMD than after smartphone use (all *p*’s ≤ 0.018).

**Table 1:** Comparison of subjective symptoms after playing games on a virtual reality smartphone-based head-mounted display (VR SHMD) or smartphone.

Symptom	VR SHMD	Smartphone	*p* value*
**Dry eye symptoms**			
Burning	1.0 (2.0)	1.0 (1.0)	0.018
Feeling of a foreign body	0.0 (2.0)	0.0 (1.0)	0.017
Excessive blinking	2.0 (3.0)	2.0 (2.0)	0.177
Tearing	0.5 (3.0)	0.0 (1.0)	0.002
Dryness	2.0 (2.0)	2.0 (2.0)	0.518
Tingling	2.0 (2.0)	1.0 (2.5)	0.094
Increased sensitivity to light	1.0 (2.0)	0.0 (1.0)	0.840
**Visual disturbance**			
Blurred vision	1.0 (2.0)	1.0 (2.0)	0.338
Double vision	0.0 (0.0)	0.0 (1.0)	0.894
Difficulty focusing for near vision	0.0 (0.0)	0.0 (1.0)	0.013
**Neurological symptoms**			
Headache	2.5 (3.0)	1.0 (2.0)	0.001
Dizziness	2.5 (2.0)	1.0 (1.0)	< 0.001
Nausea	1.0 (3.0)	0.0 (1.0)	< 0.001
Total score	18.0 (17.0)	12.0 (8.0)	< 0.001

Data presented as median (interquartile range).

*Wilcoxon-signed rank test and adjusted using the Benjamini–Hochberg procedure.

Table 2 summarizes the changes in choroidal thickness before and after use of the VR SHMD or smartphone. There were no significant changes in the choroidal thickness measured at the five points (subfoveal, nasal 1-mm, nasal 3-mm, temporal 1-mm, and temporal 3-mm) and in the mean choroidal thickness.

**Table 2:** Changes in choroidal thickness after playing games on a virtual reality smartphone-based head-mounted display (VR SHMD) or smartphone.

Variable	VR SHMD			Smartphone		
	Pre	Post	*p *value*	Pre	Post	*p *value*
**Dominant eye**						
Subfoveal CT	304 (111.3)	318 (128.3)	0.793	307 (119.0)	291 (135.0)	0.919
1-mm zone nasal CT	275 (120.8)	300 (125.3)	0.318	272 (119.0)	272 (137.0)	0.957
1-mm zone temporal CT	175 (118.0)	187 (134.8)	0.997	189 (128.0)	196 (107.0)	0.889
3-mm zone nasal CT	309 (107.8)	313 (109.3)	0.157	307 (103.0)	309 (146.0)	0.788
3-mm zone temporal CT	313 (109.5)	320 (107.0)	0.707	295 (114.0)	298 (124.0)	0.453
Macular CT overall	279 (78.1)	283 (111.3)	0.725	278.4 (119.6)	272.8 (101.4)	0.889
**Non-dominant eye**						
Subfoveal CT	336 (155.3)	319 (129.3)	0.431	321 (138.0)	317 (127.0)	0.775
1-mm zone nasal CT	301 (151.0)	291 (144.0)	0.297	289 (120.8)	281 (136.0)	0.788
1-mm zone temporal CT	167 (90.0)	178 (110.5)	0.188	169 (84.0)	177 (88.0)	0.969
3-mm zone nasal CT	344 (128.3)	339 (111.0)	0.467	336 (103.0)	322 (125.0)	0.880
3-mm zone temporal CT	331 (114.8)	316 (98.5)	0.687	325 (114.0)	315 (109.0)	0.246
Macular CT overall	292.4 (125.9)	286.2 (97.9)	0.339	288.2 (118.0)	284.4 (92.2)	0.619

Data presented as the median (interquartile range) unless otherwise indicated. CT, choroidal thickness.

*Wilcoxon-signed rank test and adjusted using the Benjamini–Hochberg procedure.

Table 3 shows the correlations between the baseline data (refractive error, accommodative amplitude and lag, NPA, NPC, and ocular deviation) and the changes in these parameters after use of the VR SHMD based on Spearman’s correlation test. There were positive correlations between the baseline values and the changes in accommodative amplitudes in both eyes (dominant eye: r = 0.437, *p* = 0.001; non-dominant eye: r = 0.284, *p* = 0.039). The baseline accommodative amplitudes and changes in the accommodative lag of the corresponding eye showed a positive correlation (dominant eye: r = 0.440, *p* = 0.001; non-dominant eye: r = 0.321, *p* = 0.019). The baseline NPC values and accommodative lag of the non-dominant eye exhibited a negative correlation with changes in the accommodative lag of the non-dominant eye (baseline NPC: r = − 0.301, *p* = 0.028; baseline accommodative lag of the non-dominant eye: r = − 0.434, *p* = 0.001). The baseline NPA and NPC values exhibited a negative correlation with changes in the NPC (baseline NPA of the dominant eye: r = − 0.448, *p* = 0.001; baseline NPA of the non-dominant eye: r = − 0.400, *p* = 0.002; baseline NPC: r = − 0.275, *p* = 0.037). The amounts of exophoric deviation at near and far distances in the baseline measurements demonstrated a positive correlation with the changes in exophoric deviation at a far distance (baseline far distance exophoric deviation: r = 0.328, *p* = 0.013; baseline near distance exophoric deviation: r = 0.310, *p* = 0.019).

**Table 3:** Bivariate correlations between baseline and changed visual parameters.

Baseline value		Changing value										
		AR, D	AR, ND	NPA, D	NPA, ND	Acc. Amp, D	Acc. Amp, ND	Acc. Lag, D	Acc. Lag, ND	NPC	X	X’
AR, D	r	− 0.082		− 0.049		0.025		− 0.098		0.023	− 0.048	0.117
	P	0.538		0.717		0.861		0.483		0.867	0.724	0.387
AR, ND	r		− 0.128		− 0.069		− 0.029		0.134	0.034	0.017	0.120
	P		0.338		0.605		0.839		0.339	0.802	0.899	0.374
NPA, D	r	0.254		− 0.131		0.111		0.062		**− 0.448**	0.008	0.105
	P	0.054		0.327		0.428		0.657		0.001*	0.955	0.439
NPA, ND	r		0.165		0.050		0.161		− 0.006	**− 0.400**	0.030	0.032
	P		0.215		0.707		0.249		0.968	0.002*	0.826	0.814
Acc.Amp, D	r	− 0.091		− 0.093		**0.437**		**0.440**		0.118	0.028	− 0.220
	P	0.518		0.507		0.001*		0.001*		0.400	0.844	0.118
Acc.Amp, ND	r		− 0.158		− 0.110		**0.284**		**0.321**	0.136	− 0.082	0.002
	P		0.259		0.434		0.039*		0.019*	0.336	0.562	0.987
Acc.Lag, D	r	− 0.182		0.032		0.107		− 0.268		− 0.153	− 0.109	− 0.254
	P	0.192		0.818		0.447		0.052		0.274	0.443	0.069
Acc.Lag, ND	r		0.187		− 0.033		0.269		**− 0.434**	− 0.058	− 0.135	− 0.033
	P		0.181		0.817		0.051		0.001*	0.682	0.340	0.815
NPC	r	0.031	0.065	0.194	0.209	− 0.039	− 0.151	− 0.047	**− 0.301**	**− 0.275**	0.056	− 0.066
	P	0.816	0.628	0.144	0.115	0.780	0.280	0.737	0.028*	0.037*	0.681	0.624
X	r	0.099	− 0.130	0.042	0.121	− 0.185	− 0.160	0.258	0.159	0.017	**0.328**	− 0.187
	P	0.465	0.334	0.758	0.368	0.189	0.258	0.065	0.260	0.898	0.013*	0.165
X’	r	0.109	0.150	0.167	0.121	0.162	− 0.007	0.037	0.077	0.247	**0.310**	− 0.024
	P	0.419	0.264	0.216	0.370	0.252	0.960	0.793	0.587	0.064	0.019*	0.860

D, dominant eye; ND, non-dominant eye; AR, Auto-refraction without glasses; Acc. amp, Accommodative amplitude; X, exophoric deviation at far target, X’, exophoric deviation at near target; NPA, near point accommodation; NPC, near point convergence.

*Statistically significant value using Spearman’s rho correlation test and adjust using the Benjamini–Hochberg procedure.

## Discussion

Currently, the proportion of individuals who use VR as well as their playing time are increasing^1,2^. The use of VR for educating children has also increased; however, very little research has been conducted on the effects of VR HMD on the eyes^3,4,14^. In previous studies, a relatively short VR running time of approximately 30 min was used, and the results were inconsistent^3,4^. In our previous study, we could not find any refraction changes after the use of VR SHMD for 30 min, but NPA and NPC receded after an immersive mode VR gaming session. We thought that the use of VR for more than 1 h was necessary to confirm the effects of VR SHMD on visual parameters and applied it in the current study. We set 2 h for the VR session because we judged that any time less than 2 h was insufficient to induce changes in ocular parameters for comparison with a smartphone. We had hypothesized that the use of VR SHMD for a long time would affect the refraction. However, there were no changes in refraction after 2 h of VR SHMD use. The changes in visual parameters such as NPA, NPC, stereopsis, and the exophoric deviation at the near distance were presented after VR SHMD use, whereas only the NPA of the dominant eye receded after smartphone use.

Recently, many VR HMDs are commercially available that have various types with different resolutions of the display, and positional tracking systems. They also have different optical design such as Fresnel/traditional hybrid, dual aspheric lens, and panoramic lens^15^. However, these VR HMDs have similar basic principles that usually use a stereoscope. Three-dimensional stereo images can be obtained from binocular disparity, which is defined as the difference in the image location seen by both eyes. VR images inevitably accompany image disparity, and could activate an accommodative response and convergence-accommodation causing a change in accommodation^16–18^. However, in the VR environment, the actual accommodative target was fixed; thus, there was a possibility of fatigue due to the excessive activation of accommodative adaptation^17^. As a result, the visual parameters, including the NPA, NPC, stereopsis, and exophoric deviation, significantly worsened after 2 h of VR SHMD use.

The correlation analysis of the visual parameters between the baseline and the changes in the parameters after VR use showed paradoxical results. Participants with a receded NPC and higher accommodative lag were more likely to exhibit reduced changes in accommodative lag. Sreenivasan et al.^19^ reported that retinal image quality is better when the accommodative lag is higher, because the depth of field is structurally or functionally wider in individuals with a higher accommodative lag, which is paradoxical. This may reduce ocular fatigue that is induced by a rapid accommodation response. On the same principle, participants with a lower accommodation amplitude showed a lesser variation in the accommodation lag after VR use. Similarly, participants with receded NPA and NPC prior to the experiment showed lesser changes in the NPC because they experienced less fatigue after 2 h of VR SHMD use in our study. Although there was no statistical significance, the eyes tended to show a hyperopic shift tendency after VR SHMD use. As a result, the accommodative lag might have decreased. Furthermore, a higher accommodative lag may not have worsened because of the ceiling effect. In our previous study, we found that 30 min of VR SHMD use increased the accommodative lag in subjects with exophoria^5^. However, there was no correlation between exophoric deviation and accommodative lag in this study. This result may be explained by the fact that this study did not use severe immersive mode gaming and allowed participants to take a 3-min break after VR SHMD uses before the measurement of the visual parameters.

Participants with a larger exophoric deviation showed a greater tendency toward worsening of exophoria at a far distance after VR SHMD use. The changes in exophoria at a far distance showed a positive correlation with the magnitude of baseline exophoria, but those at a near distance did not. One possible explanation for this finding is that participants with a large exophoric deviation at a far distance may have decreased vergence adaptation and thereby causing them to have increase their convergence effort^9,20,21^. This greater convergence effort could induce more ocular fatigue. Increases in exophoric deviations after VR use only at a far distance may occur due to the convergence stimulus, which can compensate for ocular fatigue, as it is weaker at a far distance than at a near distance.

Myopia is a common eye problem among adolescents and its prevalence is exceptionally high in the Asian population^22^. Peripheral defocus and reduced time outdoors may have been shown to predispose patients to myopia.^23,24^ Factors such as accommodative lag can induce myopia by creating a defocused environment, and choroidal thickness may also be related to myopic progression owing to blood flow in the eyeball and growth factors^12,23,25,26^. Some studies have suggested that a VR environment could control these factors and prevent myopia, but no experiment has been conducted to verify this hypothesis^27^. Turnbull et al.^4^ reported that the choroid was thickened after 40-min VR trials in indoor and outdoor environments. They suggested that VR HMD use could be associated with choroidal thickening after myopic defocus, which is associated with the slowing of eye growth and a reduction in myopic progression^4^. However, in our study, there were no changes in choroidal thickness or refraction after 2 h of VR SHMD use. Another report showed that a temporary, transient myopic shift could have occurred after VR use^3^. Further studies with larger populations that include adolescents may be needed to confirm the existence of changes in choroidal thickness and refraction after VR use.

On the questionnaire, the participants reported more subjective dry eye symptoms after the 2-h VR SHMD use than after the smartphone use. The VR SHMD used in this study was assembled with a smartphone, but a blue-light filter was not applied. Previous reports stated that the aggravation of subjective symptoms and induction of oxidative stress in tears and the ocular surface are possibly higher after smartphone use than after computer use^28,29^. The authors attributed this effect to the distance between the screen and the eyes.^28^ VR also uses the same smartphone display and is closer to the user’s eyes; therefore, there is a possibility that it affects the tear film and ocular surface. However, a recent study suggested that VR HMD have a positive influence on dry eyes because of the effect of heat^30^. In our study, questionnaires were employed, but the ocular surface and tear film were not examined. In addition, the VR HMD and the duration of their use differed in each study; therefore, it is difficult to compare the results of previous studies to those of our study. Neurological symptoms including headache, dizziness, and nausea were more severe after the use of a VR SHMD than after the use of a smartphone. In the real world, accommodation and convergence occur harmoniously. However, in VR environments, accommodation is fixed to a single depth of field at a distant point, whereas convergence is constantly induced. This accommodation-convergence conflict is known to cause fatigue and 3D asthenopia^13,31–33^. Fatigue induced by accommodation-convergence conflict could lead to the development of more severe neurologic symptoms in VR gamers than in smartphone gamers. In this study, we measured visual parameters after a 3-min break with the eyes closed. We believe that this break may have minimized the effects of the neurologic symptoms on visual parameters, the measurement of which required the attention of the participants.

This study has several limitations. First, determining the participants who were more susceptible to the impact of VR SHMD use on visual parameters is important for identifying populations requiring more attention when using VR SHMD. However, we included only a specific population (i.e., 20 to 39 years of age, only one ethnicity) and could not include adolescents because of safety issues regarding extended VR SHMD use. Second, we could not use an additional eye-tracking instrument to measure actual ocular movement and pupillary changes. Third, we used VR SHMD that was released in 2015. New VR HMDs, which have a high resolution and refresh rate and a wide field of view, have been developed and released. We cannot say that our results would be reproduced in new and other types of VR HMD. Fourth, we used different games for VR SHMD and smartphones. VR games used in this study were for VR only, and the same game could not be found for the smartphone at that time. We chose a puzzle game for the smartphone, which is familiar and can be played for a long time. We tried to match it as much as possible by choosing a VR game that has a comfortable grading system and includes puzzle games. Further experiments using various VR SHMD and different durations per session while controlling the game contents will be needed. Finally, the participants were allowed to rest for less than 5 min during the experiment, if they desired. This may have introduced a bias, but we believe that allowing a short break was more likely to emulate the actual use of VR SHMD.

In conclusion, the 2-h use of VR SHMD did not affect refraction but affected accommodation, convergence, exodeviation, and subjective symptoms. Participants with larger exodeviation showed a greater tendency toward worsening of exophoria at a far distance after VR SHMD use. Participants who had poor accommodation and convergence power were paradoxically more resistant to changes in visual parameters. This is the first study to evaluate the effects of prolonged use of VR SHMD on visual parameters.

## Data Availability

The datasets generated during the current study are available from the corresponding author on reasonable request.
